# Sequence and phylogenetic analysis of the complete mitochondrial genome for Hepu mitten crab (*Eriocheir hepuensis*) from Nanjiujiang River basin

**DOI:** 10.1080/23802359.2019.1688117

**Published:** 2019-11-08

**Authors:** Cheng Zhang, Qingqing Li, Qingguo Meng, Wen Wang, Yongxu Cheng, XuGan Wu

**Affiliations:** a Key Laboratory of Freshwater Aquatic Genetic Resources, Ministry of Agriculture, Shanghai Ocean University, Shanghai, China;; b Shanghai Collaborative Innovation Center for Aquatic Animal Genetics and Breeding, Shanghai Ocean University, Shanghai, China;; c Jiangsu Key Laboratory for Biodiversity & Biotechnology and Jiangsu Key Laboratory for Aquatic Crustacean Diseases, College of Life Sciences, Nanjing Normal University, Nanjing, PR China;; d National Demonstration Centre for Experimental Fisheries Science Education, Shanghai Ocean University, Shanghai, China

**Keywords:** *Eriocheir hepuensis*, mitochondrial genome, taxonomic classification, phylogenetic

## Abstract

Taxonomic classification of *Eriocheir hepuensis* was ambiguous, and it has long been controversial. In this study, the whole mitochondrial genome of *E. hepuensis* was determined to be 16,397 bp, including 13 protein-coding genes, 22 transfer RNA genes, 2 ribosomal RNA genes, and 1 control region. A total of 20 intergenic gaps were detected, and the AT content of whole mitochondrial genome was 71.78%. Phylogenetic analysis confirmed that the evolutionary relationship of *E. hepuensis*, *E. sinensis*, and *E. japonica* are most likely to be three species with the same taxonomic status. The whole mitogenome of this species will be useful for the future animal evolutionary, phylogenetic relationship, phylogeny and genomic studies in the genus *Eriocheir*.

Hepu mitten crab (*Eriocheir hepuensis*) belongs to the genus: Decapoda, Varunidae, *Eriocheir*. But its higher classifications were ambiguous, and taxonomic boundaries between genera and species, and species and subspecies of *E. hepuensis* have been controversial (Li and Li 1999; Li and Zou 1999; Tang et al. 2003; Wang et al. 2008). In the present study, specimen of *E. hepuensis* (Wu, HP7) was collected from November to December 2017 by fishermen in Nanliujiang River basin (109.04°E, 21.39°N), and it was deposited in Key Laboratory of Freshwater Aquatic Genetic Resources, Ministry of Agriculture, Shanghai Ocean University, Shanghai, China. The total genomic DNA was extracted using phenol-chloroform protocol, and the complete mitochondrial genome (mtDNA) was determined by using High-throughput Illumina Sequencing technology, and proofread by Sanger method. The location of protein-coding genes (PCGs), transfer RNA (tRNA) genes and ribosomal RNA (rRNA) genes were identified using the MITOS Web Server (Bernt et al. 2013). The annotated genomic sequence has been submitted to GenBank with the accession number MK159104.

In total, the complete mtDNA of *E. hepuensis* was 16,397 bp in length. The overall A + T content of the whole mtDNA was 71.78%. The complete mtDNA contained 13 PCGs, 22 tRNA genes, two rRNA genes, and one control region. Among the 37 genes, four PCGs (*ND1*, *ND4*, *ND4L*, and *ND5*), eight tRNA genes (*tRNA^Pro^*, *tRNA^Leu(CUN)^*, *tRNA^His^*, *tRNA^Val^*, *tRNA^Gln^*, *tRNA^Cys^*, *tRNA^Tyr^*, and *tRNA^Phe^*), and two rRNA genes (*12S rRNA*, *16S rRNA*) were on the light strand, and the remaining 23 genes were on the heavy strand. All 13 PCGs from the complete mtDNA were initiated by the typical start codon ATN (ATA for *ND1* and *ND6* genes, ATC for *ND2* and *ND3* genes, ATC for *ND3* gene, ATT for *ATP6*, ATG for *ATP8*, *COX I*, *COX II*, *COX III*, *Cyt b*, *ND4*, *ND4L*, and *ND5* genes and ATT for *ND6* gene). The typical termination codons (TAA or TAG) were detected in 10 PCGs (TAA for *ATP6*, *ATP8*, *COX II*, *COX III*, *ND1*, *ND3*, *ND4*, *ND4L*, and *ND6* genes, TAG for *ND2* and *ND5* genes). The remaining two genes (*COX I* and *Cyt b*) were ended by incomplete stop codons (T––). All tRNA genes, ranged from 63 bp to 73 bp, and a total of 29 base mismatches were detected. Among them, there are one mismatch number of A–A base pairs, two mismatches of A–C base pairs, two mismatches of U–U base pairs, and 24 mismatches of U–G base pairs. The *12S rRNA* is 827 bp, while the *16S rRNA* is 1336 bp, and they were located between *tRNA^Leu (CUN)^* and *tRNA^His^*. The 952 bp control region was located in *tRNA^Val^* and *tRNA^Gln^*. A total of 20 intergenic gaps were detected in the complete mtDNA of *E. hepuensis*, and A + T content of the control region was 83.61%. The phylogenetic relationship was estimated using the maximum likelihood method. The results showed that *E. hepuensis*, *E. sinensis*, and *E. japonica* are most likely to be three species with the same taxonomic status (Figure 1).

**Figure 1.**
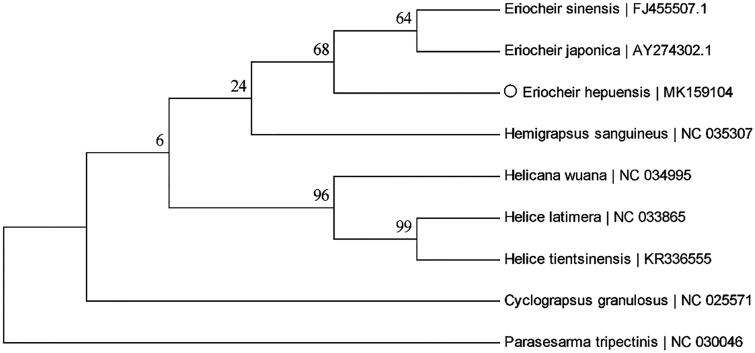
The Maximum Likelihood tree inferred from 13 PCGs of nine species. Numbers at the branches indicated the bootstrapping values with 1000 replications. GenBank accession numbers are on the right side of the vertical line. ‘Hollow circle’ represents a sequence in this study.
